# Specific NOTCH1 antibody targets DLL4-induced proliferation, migration, and angiogenesis in *NOTCH1*-mutated CLL cells

**DOI:** 10.1038/s41388-019-1053-6

**Published:** 2019-10-15

**Authors:** Mónica López-Guerra, Sílvia Xargay-Torrent, Patricia Fuentes, Jocabed Roldán, Blanca González-Farré, Laia Rosich, Elisabeth Silkenstedt, María J. García-León, Eriong Lee-Vergés, Neus Giménez, Ariadna Giró, Marta Aymerich, Neus Villamor, Julio Delgado, Armando López-Guillermo, Xose S. Puente, Elias Campo, María L. Toribio, Dolors Colomer

**Affiliations:** 1 Experimental Therapeutics in Lymphoid Malignancies Group, Institut d’Investigacions Biomèdiques August Pi i Sunyer (IDIBAPS), Barcelona, Spain; 2 Hematopathology Section, Hospital Clínic, Institut d’Investigacions Biomèdiques August Pi i Sunyer (IDIBAPS), Barcelona, Spain; 3 Centro de Investigación Biomédica en Red de Cáncer (CIBERONC), Barcelona, Spain; 4 Centro de Biología Molecular Severo Ochoa, Consejo Superior de Investigaciones Científicas, Universidad Autónoma de Madrid, Madrid, Spain; 5 Universitat de Barcelona, Barcelona, Spain; 6 Department of Internal Medicine III, University Hospital, Ludwig Maximilian University, Munich, Germany; 7 Institut d’Hématologie-Immunologie, INSERM U1109, Strasbourg, France; 8 Hematology Department, Hospital Clínic, Institut d’Investigacions Biomèdiques August Pi i Sunyer (IDIBAPS), Barcelona, Spain; 9 Departamento de Bioquímica y Biologí, a MolecularInstituto Universitario de Oncología, Universidad de Oviedo, Oviedo, Spain

**Keywords:** Leukaemia, Cancer

## Abstract

Targeting Notch signaling has emerged as a promising therapeutic strategy for chronic lymphocytic leukemia (CLL), particularly in *NOTCH1*-mutated patients. We provide first evidence that the Notch ligand DLL4 is a potent stimulator of Notch signaling in *NOTCH1*-mutated CLL cells while increases cell proliferation. Importantly, DLL4 is expressed in histiocytes from the lymph node, both in *NOTCH1*-mutated and -unmutated cases. We also show that the DLL4-induced activation of the Notch signaling pathway can be efficiently blocked with the specific anti-Notch1 antibody OMP-52M51. Accordingly, OMP-52M51 also reverses Notch-induced *MYC, CCND1*, and *NPM1* gene expression as well as cell proliferation in *NOTCH1*-mutated CLL cells. In addition, DLL4 stimulation triggers the expression of protumor target genes, such as *CXCR4*, *NRARP*, and *VEGFA*, together with an increase in cell migration and angiogenesis. All these events can be antagonized by OMP-52M51. Collectively, our results emphasize the role of DLL4 stimulation in *NOTCH1*-mutated CLL and confirm the specific therapeutic targeting of Notch1 as a promising approach for this group of poor prognosis CLL patients.

## Introduction

Activating mutations in *NOTCH1* have emerged as one of the most frequent somatic alterations in chronic lymphocytic leukemia (CLL), affecting up to 10–15% of patients at diagnosis [1, 2]. Clinically, *NOTCH1*-mutated patients have features associated with adverse prognosis and high risk of transformation [2–4]. The majority of these mutations abrogate the PEST domain and generate a truncated protein that accumulates in the cell and activates downstream signaling [2]. More recently, recurrent mutations in the noncoding 3’UTR of *NOTCH1* have been identified in ~3% of CLL patients, which cause aberrant splicing events that lead to the loss of the PEST domain and increase Notch1 activity [5]. The functional effect of the different types of *NOTCH1* mutations has been extensively studied in T-acute lymphoblastic leukemia (T-ALL), where most Notch1 alterations affect the heterodimerization domain of the receptor and lead to a constitutive ligand-independent Notch activation [6]. In contrast, both PEST and 3’UTR mutations described in CLL are considered as weak *NOTCH1* mutations, not oncogenic by themselves, and are ligand-dependent [5, 6].

Jagged and Delta-like ligands interact with Notch receptors to induce their cleavage and nuclear translocation of the intracellular domain. Once in the nucleus, Notch activates the transcription of target genes including *HES1* and *MYC*. Notch1 target genes regulate key biological processes such as development, cell differentiation, cell-fate decisions, proliferation, and apoptosis [7]. In CLL, autocrine and paracrine mechanisms of Notch activation have been suggested, as both tumor CLL lymphocytes as well as cells from the microenvironment express Notch ligands, particularly Jagged1 and Jagged2 [8, 9]. However, knowledge about the role of Delta-like ligands in CLL is still limited. Although *NOTCH1* mutations have a prominent role in the pathogenesis of CLL, alternative nonmutational mechanisms of *NOTCH1* activation have been recently described in CLL [10], indicating that the constitutive activation of the pathway in this leukemia is more frequent than it was first estimated by the incidence of the main recurrent genetic lesions. For this reason, targeting Notch signaling has emerged as a promising therapeutic strategy for CLL, with the hypothesis that its inhibition might also provide an improvement in the efficacy of the standard chemotherapy. Our group previously reported the antitumor effect of the γ-secretase inhibitor (GSI) PF-03084014 in combination with fludarabine in CLL cells carrying *NOTCH1* mutations [11]. Similarly, a marked in vitro resistance to drug-induced apoptosis in CLL cells harboring *NOTCH1* mutations has been reported, which may be abrogated by GSI [8]. Moreover, the combination of PF-03084014 and fludarabine is able to reduce angiogenesis and CXCL12-induced responses in *NOTCH1*-mutated CLL cells, in particular those related to tumor migration and invasion [11]. Although preclinical and clinical data using GSIs are encouraging, the main limitations of GSI treatment include nonselectivity and gastrointestinal toxicity [12]. In the last years, antibodies against the specific Notch receptors have been developed, with the idea of avoiding these undesirable side effects [12]. Targeting the individual Notch1 receptor has shown promising preclinical results in T-ALL [13–15], indicating the need to explore this therapeutic strategy in other models of lymphoid malignancies. In this context, the aim of the present study was to define the role of Delta-like ligand stimulation in *NOTCH1*-mutated CLL cells as well as to explore the therapeutic disruption of this signaling with a specific anti-NOTCH1 antibody.

## Results

### DLL4 is a potent stimulator of Notch signaling and proliferation in *NOTCH1*-mutated CLL

**Fig. 1:**
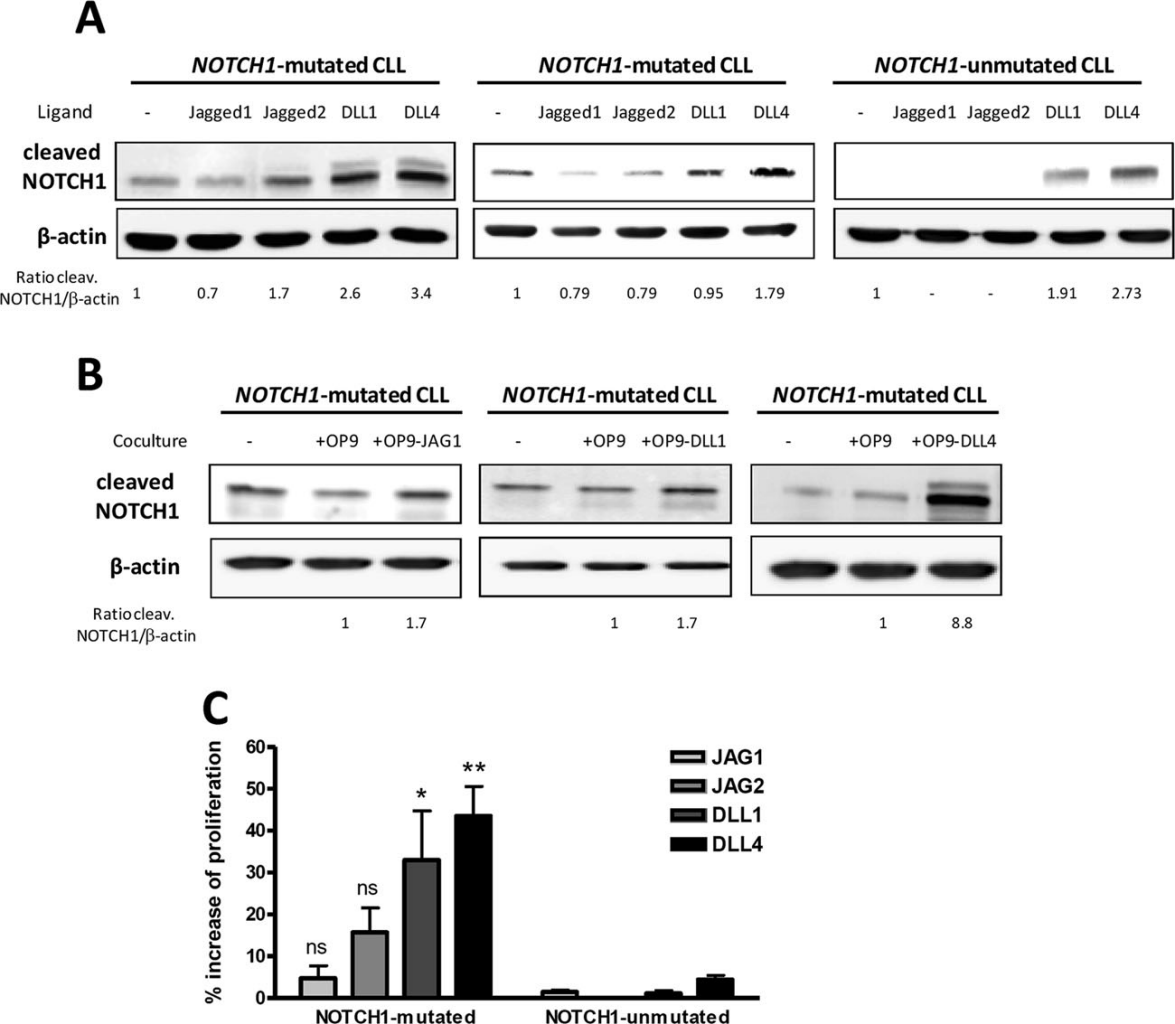
DLL4 is a potent stimulator of Notch pathway and proliferation in *NOTCH1*-mutated CLL. Cells from *NOTCH1*-mutated and -unmutated CLL cases were stimulated with Jagged1 (JAG1), Jagged2 (JAG2), DLL1, and DLL4 (10 μg/ml) ligands (**a**) or stromal cells OP9-JAG1, OP9-DLL1, and OP9-DLL4 (**b**). Cleaved Notch1 was analyzed by Western blot at 24 h and densitometrically quantified. β-Actin was probed as a loading control. Representative cases are shown (CLL 6, 9, and 18). **c** CFSE-labeled CLL cells were incubated with ODN-2006 and IL-15 together with JAG1, JAG2, DLL1, or DLL4. Reduction of CFSE fluorescence in viable CLL cells was quantified after 6 days by flow cytometry. Graph shows the percentage of cell proliferation induction of each ligand with respect to the unstimulated control. Mean ± SEM of all the samples analyzed (*n* = 6 *NOTCH1*-mutated and *n* = 6 *NOTCH1*-unmutated). **p < 0.01; *p < 0.05, ns not significant

To evaluate the effect of Notch ligands in CLL, we first stimulated primary CLL cells with the recombinant ligands Jagged1, Jagged2, DLL1, and DLL4 at 10 μg/mL. To obtain a potent activation of Notch1 the excess of soluble ligand was not removed, indicating that most of the agonistic effect was due to the remaining soluble ligand. After 24 h we analyzed the expression of the active form of Notch1 by Western blot. As shown in Fig. 1a, cleaved Notch1 was detected at basal levels in cells from *NOTCH1*-mutated cases, and, after stimulation with soluble Notch ligands, this activated form of Notch1 increased particularly after Delta-like ligand stimulation. In *NOTCH1*-unmutated CLL, as previously described [2], no basal cleaved Notch1 was detected, although DLL4 and DLL1 were able to activate Notch1. To further confirm the potent stimulation of Notch signaling by Delta-like ligands in an immobilized model, we cocultured primary CLL cells with the stromal cells OP9 expressing the different human Notch ligands Jagged1 (OP9-JAG1), DLL1 (OP9-DLL1), and DLL4 (OP9-DLL4). After 24 h, *NOTCH1*-mutated CLL cells cocultured with OP9-DLL4 showed the strongest activation of Notch1 in comparison with the other ligands (Fig. 1b), suggesting that DLL4 could be the main ligand responsible for Notch activation in CLL both under soluble and immobilized conditions.

Next, we sought to determine whether Notch stimulation had a functional relationship with CLL proliferation. To this aim, we used a CFSE-based assay to monitor the proliferation of CLL cells after a 6-days exposure with CpG oligodeoxynucleotide (ODN-2006) and IL-15, as both factors collaborate in promoting in vitro CLL growth [16]. We showed that proliferation induction resulted to be significant only in *NOTCH1*-mutated CLL cells stimulated with Delta-like ligands, especially with DLL4 (*p* = 0.001), consistent with cleaved Notch1 expression (Fig. 1c). Interestingly, this functional effect was specific for *NOTCH1*-mutated cells and was almost undetected in unmutated cases. All these results indicated a particular sensitivity of CLL cells to DLL4 stimulation.

### DLL4 is expressed in the CLL lymph node

**Fig. 2:**
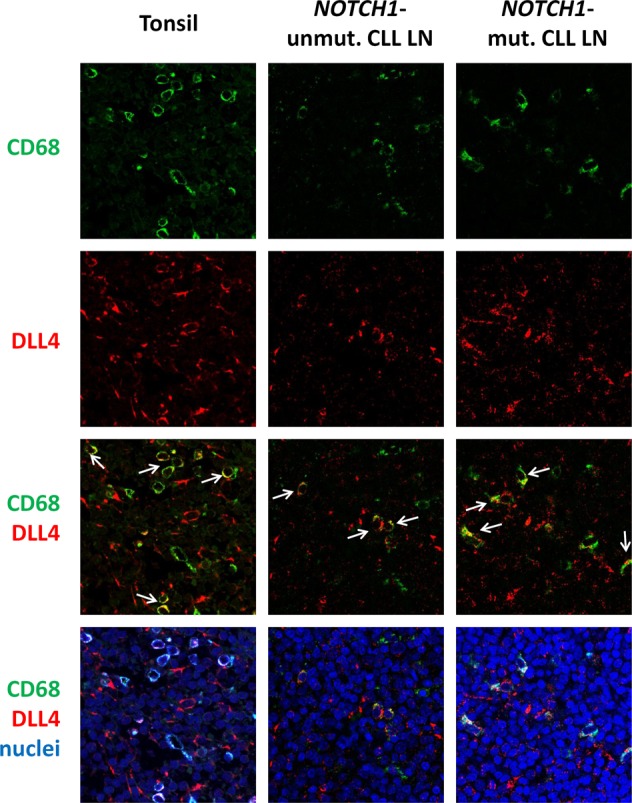
DLL4 is expressed in CLL lymph nodes. Immunofluorescence staining of tonsils and CLL LN with anti-CD68 (green) and anti-DLL4 (red). Nuclei were stained with Topro-3 (blue). White arrows indicate representative CD68+/DLL4+ cells. Pictures were taken at ×63 magnification in a confocal microscope. Images from a representative case per group are shown (CLL 11 and 19)

In view of the remarkable in vitro effect of DLL4 in *NOTCH1*-mutated CLL, we sought to characterize which cells could express this ligand and trigger the activation of Notch in vivo. We analyzed the expression of DLL4 in the different cellular populations present in lymph nodes (LN) infiltrated by CLL, using immunofluorescence staining and confocal microscopy and tonsil biopsies as controls. In tonsils, we observed coexpression of DLL4 with the monocytic marker CD68 in some histiocytes (Fig. 2). Although tissue architecture is completely effaced in CLL LN, we found that the Notch ligand DLL4 was widely expressed in the vascular endothelium (Supplemental Fig. 1) and, importantly, it was also expressed by some CD68+ cells in both *NOTCH1*-mutated and -unmutated cases (Fig. 2). All these results suggested that DLL4 is expressed in the lymph node CLL compartment, and could thus provide a specific niche for Notch activation.

### The anti-NOTCH1 antibody OMP-52M51 inhibits DLL4-induced Notch activation in CLL

**Fig. 3:**
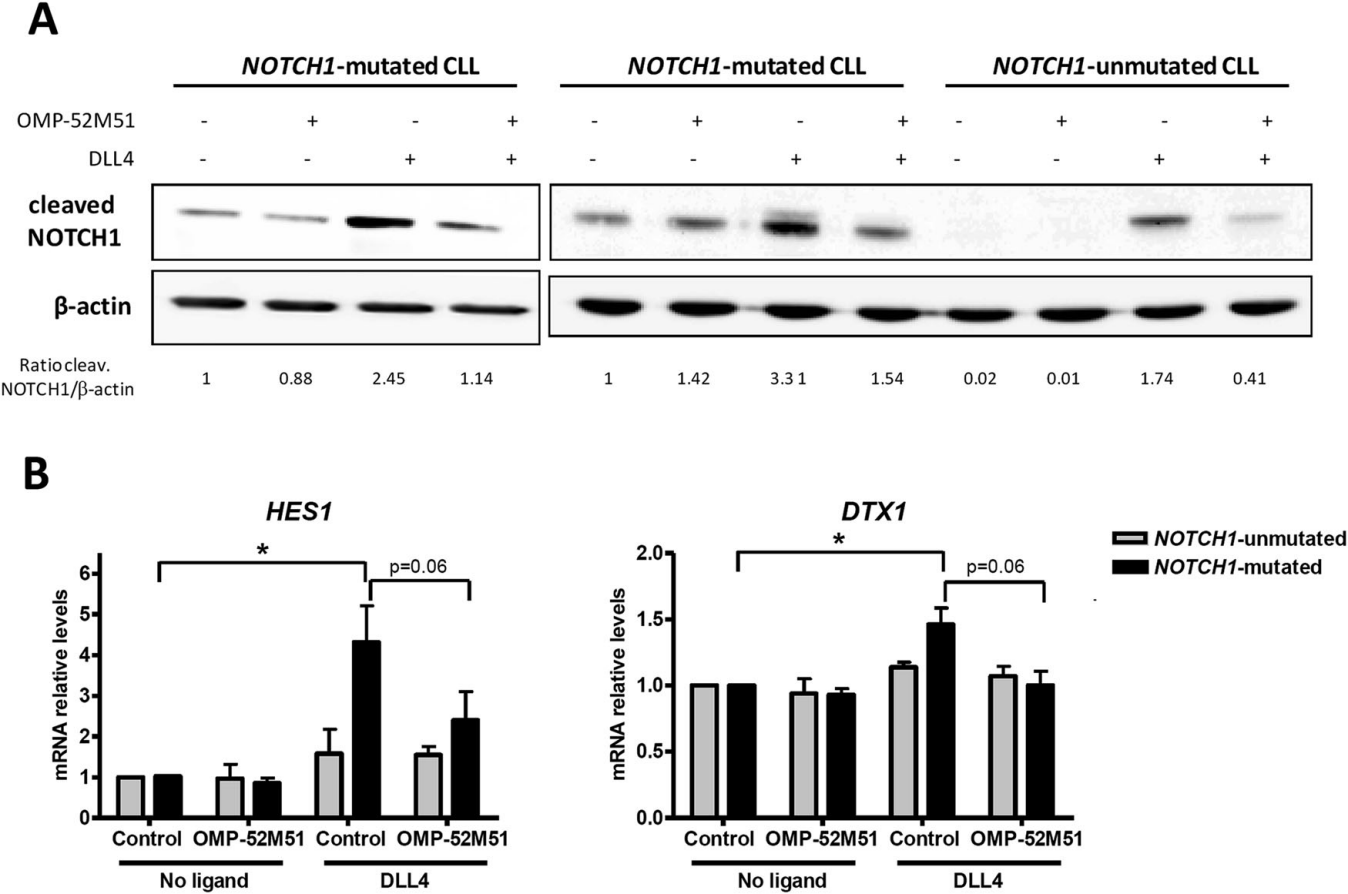
OMP-52M51 inhibits DLL4-induced Notch activation in CLL. Primary cells from *NOTCH1*-mutated and *NOTCH1*-unmutated CLL cases were pretreated for 2 h with OMP-52M51 before DLL4 stimulation (4 μg/mL) for 24 h. **a** Cleaved Notch1 was assessed by Western blot and densitometrically quantified. β-Actin was probed as a loading control. Representative cases are shown (CLL 2, 8, and 18). **b** Gene expression analysis by quantitative real time PCR of *HES1* and *DTX1*. mRNA relative levels are given as arbitrary units, using untreated condition as a reference. *Control*: isotype control. Bars represent the mean ± SEM of *NOTCH1*-mutated (*n* = 5) and -unmutated (*n* = 5) CLL cases. *p* < 0.05

To evaluate the potential therapeutic targeting of ligand-dependent Notch signaling in CLL, we considered our functional results above showing that DLL4 promoted effective Notch activation in *NOTCH1*-mutated CLL cells. Thus, primary CLL cells were treated for 2 h with the specific anti-NOTCH1 antibody OMP-52M51 before DLL4 stimulation. In all the experiments with OMP-52M51, an isotype control was added to the untreated condition. Then we monitored the modulation of cleaved Notch1 by Western blot after 24 h. In contrast to what we observed when CLL cells were stimulated by DLL4 alone, the addition of OMP-52M51 impaired Notch1 DLL4-dependent stimulation. This effect was more pronounced in cells from *NOTCH1*-mutated cases but was also observed in *NOTCH1*–unmutated ones (Fig. 3a). The treatment with OMP-52M51 only reversed the fraction of cleaved Notch1 increased with the ligand but not the basal active Notch1. Moreover, we analyzed the levels of Notch1-direct target genes after OMP-52M51 treatment and ligand stimulation. Using quantitative PCR, we found that DLL4 increased the mRNA levels of *HES1* and *DTX1* specifically in *NOTCH1*-mutated cases, and OMP-52M51 abrogated almost completely this effect after 24 h of treatment (Fig. 3b). In contrast, no transcriptional effect of DLL4 was observed in *NOTCH1*-unmutated cases either with or without antibody treatment.

All these results indicated that the activation of Notch signaling pathway induced by DLL4 in *NOTCH1*-mutated CLL is stronger than in unmutated cases and could be blocked with a specific Notch1 receptor antibody.

### OMP-52M51 inhibits DLL4-induced CLL proliferation

**Fig. 4:**
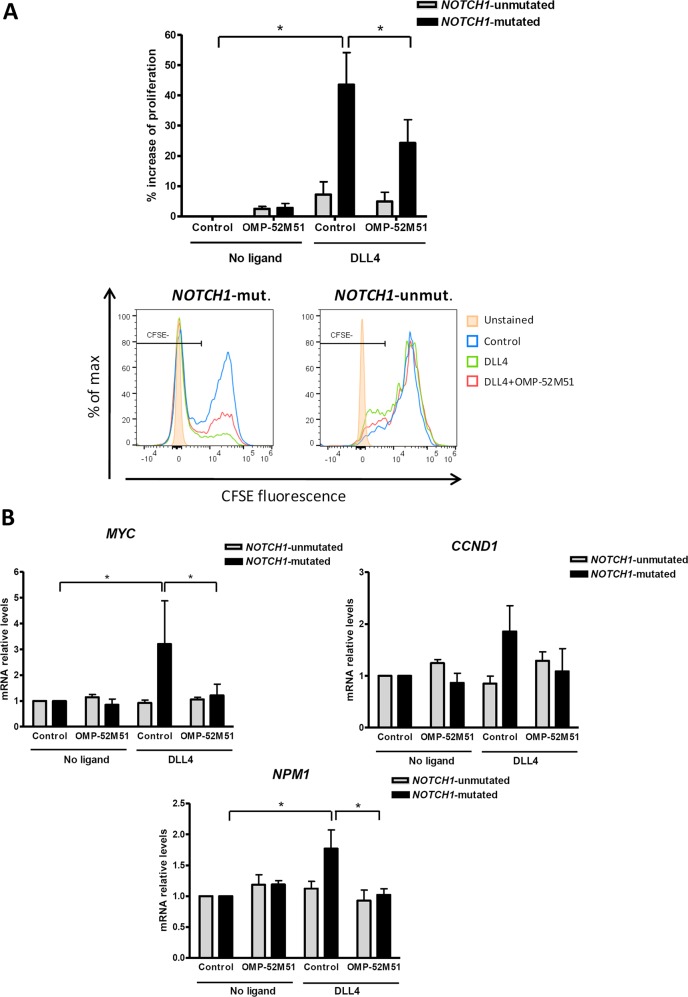
OMP-52M51 inhibits DLL4–induced proliferation. **a** CFSE-stained CLL cells were pretreated for 2 h with OMP-52M51 before DLL4 stimulation (4 μg/mL) for 6 days. Reduction of CFSE fluorescence in viable CLL cells was quantified by flow cytometry. Graph shows the percentage of cell proliferation induction with respect to the unstimulated control. Mean ± SEM of all the samples analyzed. Bottom panel shows the histograms of CFSE staining in representative CLL cases (CLL 2 and 15). **b** Cells from *NOTCH1*-mutated and *NOTCH1*-unmutated CLL cases were pretreated for 2 h with OMP-52M51 before DLL4 stimulation (4 μg/mL) for 72 h. Gene expression of *MYC, CCND1, and NPM1* was analyzed by quantitative real time PCR. mRNA relative levels are given as arbitrary units, using untreated cells as a reference. *Control*: isotype control. Bars represent the mean ± SEM of *NOTCH1*-mutated (*n* = 6) and -unmutated (*n* = 4) CLL cases. *p* < 0.05

Since one of the most impressive effects of DLL4 stimulation was the induction of cell proliferation in *NOTCH1*-mutated CLL, we were interested in exploring the ability of OMP-52M51 to antagonize this effect. Using a CFSE-based assay in long-term cultures, we found that the DLL4-induced increase in cell proliferation in *NOTCH1*-mutated CLL cells was partially but significantly blocked by OMP-52M51 (*p* < 0.05, Fig. 4a). According to these results, we investigated the modulation of three genes known to play a key role in the control of cell proliferation, *MYC, NPM1*, and *CCND1*, which have been functionally related to Notch pathway in leukemic cells [10, 17, 18]. Thus, after 72 h, DLL4 significantly upregulated *MYC* (*p* < 0.05) and *NPM1* (*p* < 0.05) in *NOTCH1*-mutated CLL cells, and *CCND1* showed a similar trend (Fig. 4b). Consistently, OMP-52M51 inhibited the DLL4-induced *MYC and NPM1 (p* < 0.05), and tended to reduce *CCND1* gene expression, specifically in CLL cells carrying *NOTCH1* mutation (Fig. 4b). These results suggested that Notch1 signaling upregulates cell proliferation including *MYC* gene expression and that this axis could be therapeutically targeted with an anti-Notch1 antibody.

### DLL4 stimulation of *NOTCH1*-mutated CLL cells induces CXCR4 expression and migration that can be inhibited by OMP-52M51

**Fig. 5:**
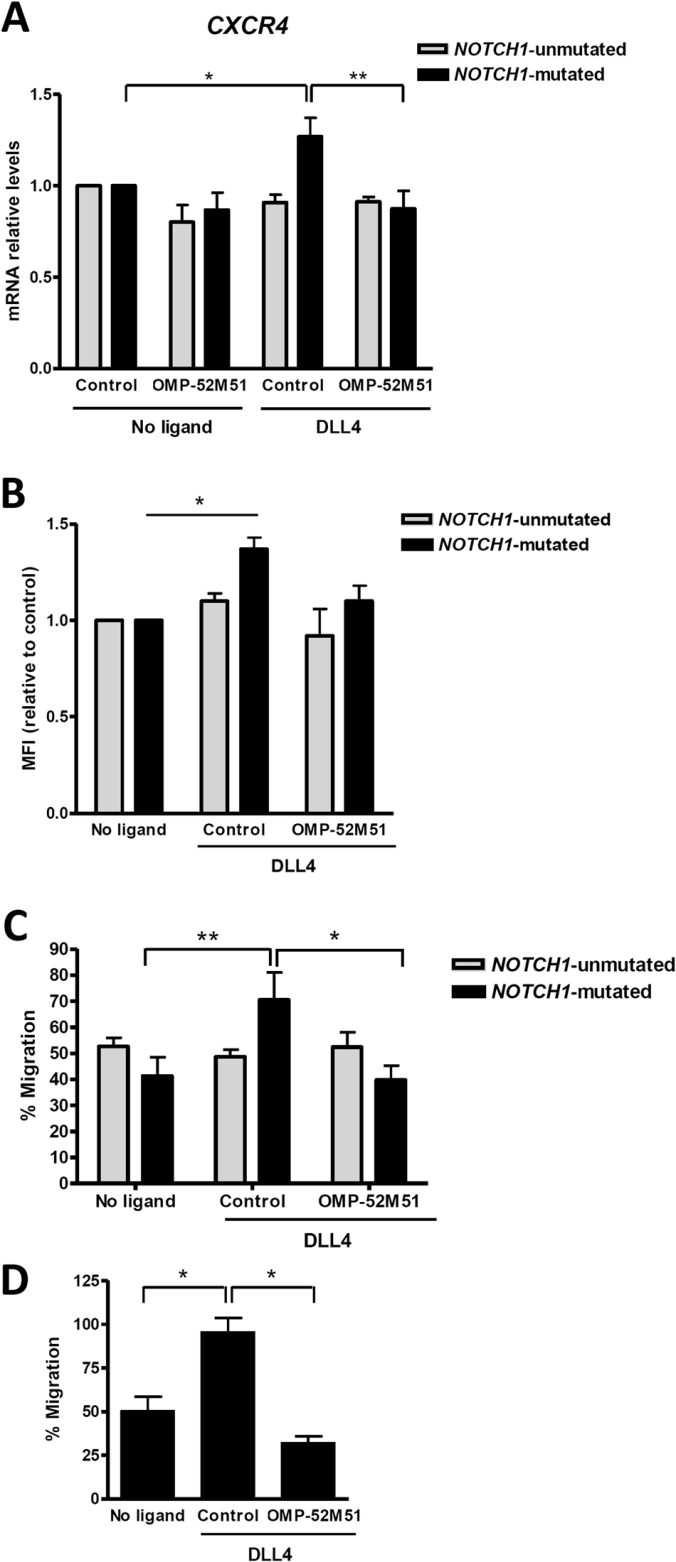
OMP-52M51 inhibits DLL4-induced CXCR4 expression and migration. Primary cells from *NOTCH1*-mutated and *NOTCH1*-unmutated CLL cases were pretreated for 2 h with OMP-52M51 before DLL4 stimulation (4 μg/mL) for 48 h. **a** *CXCR4* expression was analyzed by quantitative real time PCR. mRNA relative levels are given as arbitrary units, using untreated cells as a reference. **b** CXCR4 expression was analyzed by flow cytometry (*n* = 4 *NOTCH1*-mutated cells; *n* = 4 *NOTCH1*-unmutated cells). CXCR4 expression levels are showed based on the median fluorescence intensity (MFI) on viable cell population, using untreated cells as a reference. **c** Samples were assayed for chemotaxis toward CXCL12 (200 ng/ml). Migration is represented as the percentage of migrating cells out of total viable cells added to the transwell. *Control*: isotype control. Bars represent the mean ± SEM of *NOTCH1*-mutated (*n* = 8) and -unmutated (*n* = 4) CLL cases. *p < 0.05; **p < 0.01. **d** Samples were assayed for chemotaxis toward CXCL13 (500 ng/ml). Migration is represented as the percentage of migrating cells out of total viable cells added to the transwell. Bars represent the mean ± SEM of *NOTCH1*-mutated CLL cases (*n* = 6). *p < 0.05

Previous work reported that anti-Notch treatment effectively prevents multiple myeloma cell migration by reducing *CXCR4* expression at transcriptional level [19]. Given the importance of CXCR4/CXCL12 in CLL biology, we analyzed the effect of Notch ligand stimulation and its therapeutic targeting in this axis. With this objective, we quantified the gene expression levels of *CXCR4* by quantitative PCR and protein levels by flow cytometry and assayed CLL cell migration toward CXCL12 after 48 h of incubation with OMP-52M51 and ligand stimulation. Exposure to DLL4 upregulated *CXCR4* mRNA expression as well as protein levels specifically in *NOTCH1*-mutated CLL cells (*p* < 0.05), together with an induction of the CXCL12-induced migratory capacity of these cells (Fig. 5a–c). Importantly, the anti-Notch1 antibody significantly reversed both CXCR4 gene expression levels (*p* < 0.01) and chemotaxis in DLL4-stimulated *NOTCH1*-mutated CLL (*p* < 0.05).

Furthermore, in addition to CXCL12-induced chemotaxis, we also showed that DLL4 increased CLL cell migration toward CXCL13, another chemokine known to play a role on CLL pathogenesis [20], and similarly to CXCL12, OMP-52M51 blocked this induction in *NOTCH1*-mutated CLL (*n* = 6; Fig. 5d).

### DLL4 increases angiogenic factors in *NOTCH1*-mutated CLL that can be blocked by OMP-52M51

**Fig. 6:**
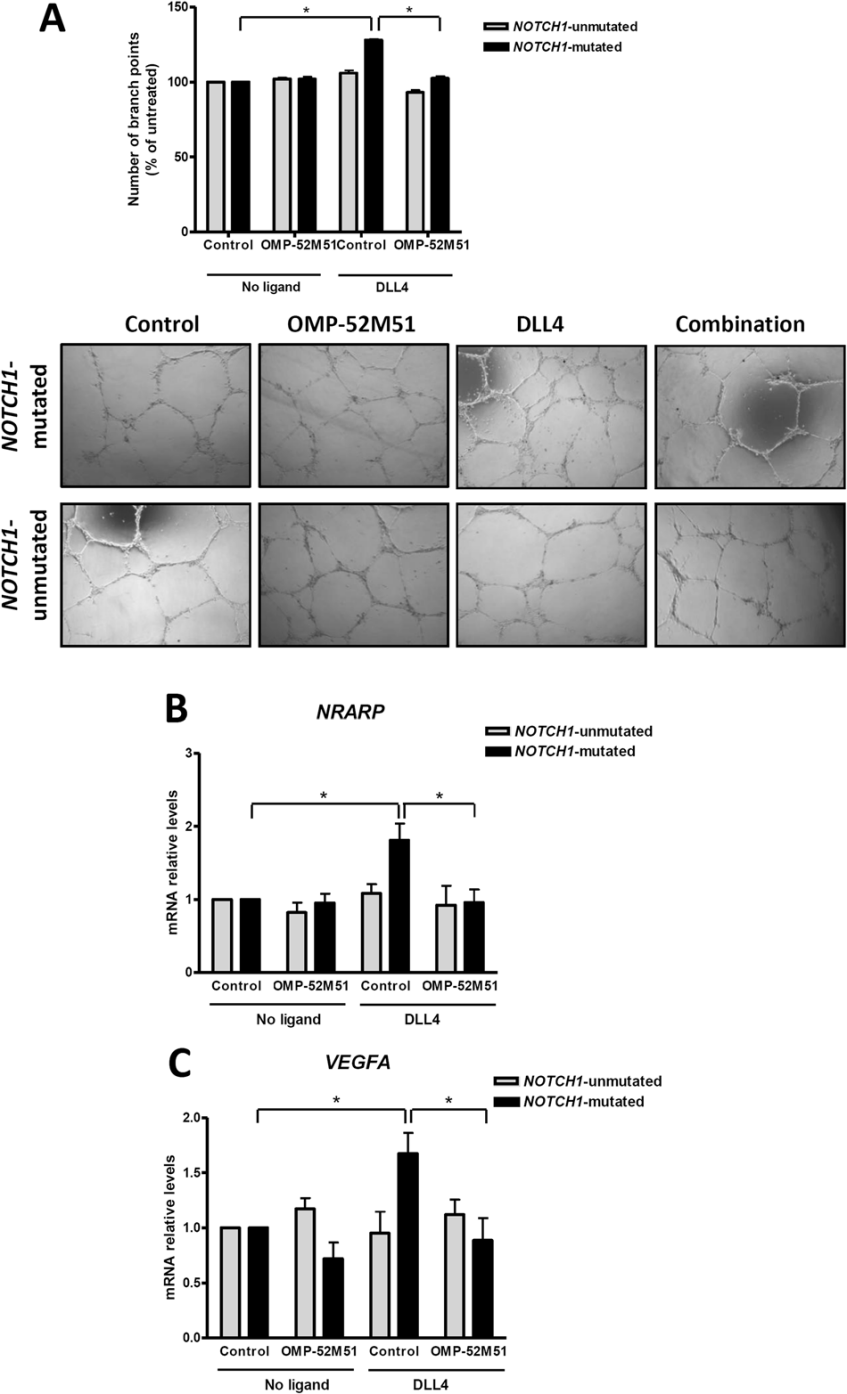
OMP-52M51 inhibits DLL4-induced angiogenesis. Primary cells from *NOTCH1*-mutated (*n* = 6) and *NOTCH1*-unmutated (*n* = 4) CLL cases were pretreated for 2 h with OMP-52M51 before DLL4 stimulation (4 μg/mL) for 72 h. **a** Supernatant from CLL cells was harvested after treatment and added to HUVEC cells for 24 h. The number of branch points was quantified as the mean of five randomly chosen fields from each well. Bars represent the mean ± SEM. *p < 0.05. Microscope images (×40 magnification) from a representative case per condition are shown (CLL 2 and 12). **b** *NRARP* and *VEGFA* expression was analyzed by quantitative real time PCR. mRNA relative levels are given as arbitrary units, using untreated cells as a reference. *Control*: isotype control

Notch pathway is involved in physiological as well as tumor angiogenesis [21]. Therefore, we sought to explore the functional link between Notch1 and angiogenesis in CLL. To this aim, we carried out HUVEC tube formation assays with the supernatants from primary CLL cells exposed to DLL4 in the presence or absence of OMP-52M51. Supernatants from *NOTCH1*-mutated cells stimulated with DLL4 had a proangiogenic effect increasing the number of branch point of HUVEC cells, whereas OMP-52M51 hampered this effect (*p* < 0.05). Again, no effect on *NOTCH1*-unmutated cases was observed, irrespective of the ligand or antibody exposure (Fig. 6a). To identify possible mediators of this effect, we explored the mRNA expression modulation of two proangiogenic candidate genes for which a functional link with Notch has been proposed: *NRARP* and *VEGF* [22–24]. Using quantitative PCR, we showed a significant upregulation of *NRARP* and *VEGFA* levels (*p* < 0.05), which was blocked by OMP-52M51 treatment in *NOTCH1*-mutated CLL cells (Fig. 6b, c). Altogether, these results suggest a functional transcriptional link between Notch1 signaling and aggressiveness-related protumor processes in CLL and that may be disrupted by a Notch-targeted strategy.

## Discussion

*NOTCH1* mutations in CLL are activating events that increase the stability of Notch1 intracellular domain [2]. However, these mutations have a weak transforming effect and are expected to be dependent on the presence of Notch ligands in the microenvironment to trigger and maintain a constitutive Notch1 activation. Accordingly, in vitro studies have shown that crosstalk between tumor CLL cells and accessory cells is required to maintain Notch signaling [8]. However, the microenvironmental cell components as well as the ligands that lead to Notch1 activation in CLL are not yet well established. On the other hand, targeting the connection between the ligand- and the receptor-presenting cell has emerged as a new therapeutic opportunity that also needs to be explored, in particular for the high-risk *NOTCH1*-mutated CLL patients. In the present work, we have attempted to address both questions using primary CLL cells from both *NOTCH1*-mutated and -unmutated cases. First, we showed that DLL4 was a potent stimulator of Notch1 signaling mainly in *NOTCH1*-mutated CLL cells, leading to the activation of some protumor genes and inducing processes that confer aggressiveness to the tumor, such as cell proliferation, migration, and angiogenesis. Second, we identified for the first time an efficient and specific strategy to target this ligand-induced Notch activation with an anti-Notch1 antibody. Our results pointed out that the functional effects of the DLL4-induced stimulation were specific of cells carrying *NOTCH1* mutations and barely occurred in unmutated cases without basal cleaved Notch1. We hypothesized that in *NOTCH1*-unmutated cells the signaling would not be sustained enough to have a substantial transcriptional and functional impact, as the wild type Notch1 protein has a rapid turnover [25]. Accordingly, *NOTCH1* mutations in the PEST domain have been suggested to increase the cleaved Notch1 half-life [2]. The effect that DLL4 could have in CLL with alternative nonmutational *NOTCH1* activation [10] needs further validation.

We first investigated the stimulation of CLL cells with the different Notch ligands Jagged1, Jagged2, DLL1, and DLL4. Our results highlighted an important role for the Delta-like ligands DLL4 and DLL1 in CLL, being DLL4 the most potent stimulator of Notch signaling in *NOTCH1*-mutated CLL cells. Interestingly, this DLL4 stimulation was accompanied by a strong increase in CLL cell proliferation and was specific for mutated cases. However, the stimulation induced by DLL1 was also remarkable and its potential effect on CLL Notch1 stimulation cannot be rule out. DLL1 and DLL4 are structurally related and could promote a similar downstream signaling, although it has been reported that the affinity of DLL4 for Notch1 is more than 10-fold tighter than that of DLL1 [26].

In contrast to what happens in other cellular models, in which soluble Notch ligands have an antagonistic effect [27–30], in CLL we showed that both soluble and immobilized DLL4 had an activating effect in Notch1 signaling. It is currently unknown whether any form of soluble (nonmembrane bound) Notch ligand is physiologically present or relevant in the CLL microenvironment. Furthermore, we provided first evidence that DLL4 was expressed in the lymph node CLL compartment, where it could promote Notch activation in vivo. In particular, DLL4 was highly expressed in the vascular endothelium, as described in other tumor models [31], as well as in accompanying histiocytes. Thus, the stimulation of Notch induced by the DLL4 in CLL could be through the interaction of CLL cells with surrounding cells from the microenvironment. These results agree with previous data suggesting that the Notch1 pathway is strongly activated in CLL LN [5, 8], which represent a relevant proliferative niche for the tumor cells [32]. Monocytes/macrophages play a key role in CLL development and progression through their reciprocal molecular interactions [33, 34]. Our results indicate that the potential role of macrophages in the stimulation of Notch signaling by DLL4 needs further validation. Accordingly, it has been recently described that in bone marrow the monocyte/macrophage- CLL cells crosstalk upregulates *NOTCH1* and *CXCR4*, among other genes, suggesting that macrophage targeting can be therapeutically exploited in CLL [35].

Among Notch targeted therapies, GSIs have been the most broadly assessed drugs in different malignancies. In CLL, first attempts to block in vitro Notch1 signaling were based on the use of these inhibitors, alone or in combination with chemotherapy [8, 11]. Although these preclinical data showed promising results in CLL *NOTCH1*-mutated cells, the nonselectivity and gastrointestinal toxicity of GSIs observed in other types of tumors emphasized the need to explore more selective strategies to inhibit Notch1. In this context, specific antibodies against the individual Notch receptors have been developed [14, 15]. Among them, OMP-52M51 is an anti-human Notch1 monoclonal antibody that showed encouraging antitumor efficacy in xenograft models of T-ALL [13]. In *NOTCH1*-mutated cells, we showed that OMP-52M51 efficiently inhibited soluble DLL4-induced Notch stimulation as well as cell proliferation. Accordingly, OMP-52M51 also reversed the Notch-induced *MYC, CCND1*, and *NPM1* gene expression. These three genes are known to play a functional role in the control of cell proliferation in leukemic cells [10, 17, 18]. In particular, *MYC* and *NPM1* have been involved in the proliferation advantages of *NOTCH1*-mutated CLL. *MYC* is a direct NOTCH1 target and a central oncogene involved in CLL progression [36], as occurs in T-ALL [37]. *NPM1* has been identified, together with other genes related to protein biosynthesis, as a targetable *MYC*-related gene that is overexpressed in *NOTCH1*-mutated CLL cells [18]. However, these conclusions are limited as a nonphysiological situation of our soluble ligand stimulation system cannot be ruled out.

We also confirmed a functional relationship between Notch1 signaling and the microenvironmental processes related to CLL aggressiveness such as cell migration and angiogenesis, a link that was previously suggested by our group [11]. This axis could be particularly relevant in *NOTCH1*-mutated cases, as they have been shown to be associated with disease progression and transformation to more aggressive forms [4]. In this line, Notch1 signaling contributes to CCL19-driven migration of CLL cells to tissues [38]. The CXCR4/CXCL12 pathway is also fundamental for CLL homing and CXCR4 expression has been related to an increased risk for lymphoid organ infiltration and poor outcome [39]. In this context, *CXCR4* has also emerged as a novel *NOTCH1* target in CLL [10]. Remarkably, we reported that soluble DLL4-induced *CXCR4* expression and migration were both inhibited by OMP-52M51 in *NOTCH1*-mutated CLL cells. In agreement, Notch inhibition effectively prevents multiple myeloma cell migration by reducing *CXCR4* expression at the transcriptional level [19]. However, as the increase of CXCR4 by DLL4 is relatively modest compared with the chemotaxis induction, other Notch1-dependent mediators should not be discarded. In this way, the same effect of DLL4 and OMP-52M521 was observed in *NOTCH1*-mutated cell migration toward CXCL13, suggesting that Notch1 signaling would lead to a general increase in *NOTCH1*-mutated CLL chemotaxis. In parallel, we showed that supernatants from *NOTCH1*-mutated CLL cells stimulated with DLL4 increased HUVEC tube formation, whereas OMP-52M51 was able to block this proangiogenic effect. This was accompanied by the transcriptional modulation of the angiogenic factors *NRARP* and *VEGFA*. It is well established that the Notch pathway is involved in physiological as well as tumor angiogenesis [21]. In acute myeloid leukemia, leukemic cells increased HUVEC tube formation through the activation of the VEGF and DLL4 pathway [40]. In this sense, our results highlight that in CLL might be also a link between DLL4 and VEGF, although the proangiogenic role of other Notch1-targets should also be considered.

In summary, our results suggest that DLL4 expressed by tumor microenvironment could activate Notch signaling in CLL. In addition, we propose a functional link between Notch1 signaling and aggressiveness-related protumor processes in CLL, which could be disrupted by specific Notch-targeting.

## Materials and methods

### Isolation and culture of cells

Primary cells from 20 CLL patients were studied (Table 1). IGHV gene mutational status and *NOTCH1* mutations were analyzed in previous sequencing studies [2, 4, 5, 41]. Primary cells were isolated from peripheral blood by Ficoll-Paque sedimentation (GE Healthcare, Little Chalfont, UK), cryopreserved and conserved within the Hematopathology collection of our institution registered at the Biobank from Hospital Clínic-IDIBAPS (R121004-094). The ethical approval for this project including the informed consent of the patients was granted following the guidelines of the Hospital Clínic Ethics Committee. Once thawed, cells were cultured at 2 × 10^6^ cells/ml in RPMI 1640 supplemented with 10% fetal bovine serum (FBS), 2 mM glutamine and 50 μg/ml penicillin-streptomycin (Life Technologies, Carlsbad, CA, USA), in a humidified atmosphere at 37 °C containing 5% carbon dioxide.

**Table 1:** Characteristics of CLL patients

Patient no.	Age at diagnosis	Gender	Binet/Rai stage	Previous treatment	%CD19/ CD5^a^	IGVH status	NOTCH1 status^b^	NOTCH1 mutation^b^	%NOTCH1-mutation	Cytogenetic alterations^c^
**CLL 1**	77	F	B/II	Fludarabine, FC	82	UNMUT	MUT	p2515fs*4	50	11q del, 13q del
**CLL 2**	45	M	A/II	No	85	UNMUT	MUT	p2515fs*4	43	13q del
**CLL 3**	62	F	C/III	2CdA, chlorambucil, CHOP-like, FCM	93	ND	MUT	p2515fs*4	>40^d^	13q del
**CLL 4**	54	M	B/II	No	90	UNMUT	MUT	p2515fs*4	70	Normal
**CLL 5**	60	F	A/0	No	87	MUT	MUT	p2515fs*4	>40^d^	13q del
**CLL 6**	54	M	A/0	No	90	UNMUT	MUT	p2515fs*4	9	Normal
**CLL 7**	69	M	A/0	No	90	UNMUT	MUT	p2515fs*4	48	trisomy 12
**CLL 8**	77	F	B/II	No	92	UNMUT	MUT	p2515fs*4	50	11q del, 13q del
**CLL 9**	77	M	A/I	Chlorambucil	86	UNMUT	MUT	p2515fs*4	53	11q del, trisomy 12
**CLL 10**	61	F	BII	No	81	ND	MUT	p2515fs*4	>40^d^	13q del
**CLL 11**	64	M	C/IV	Chlorambucil, fludarabine	65	UNMUT	MUT	p2515fs*4	>40^d^	Normal
**CLL 12**	59	F	B/II	Fludarabine, R-FCM	94	UNMUT	MUT	p2515fs*4	15	11q del, 13q del
**CLL 13**	44	M	B/II	No	97	UNMUT	UNMUT	UNMUT	–	13q del
**CLL 14**	61	M	C/III	No	96	UNMUT	UNMUT	UNMUT	–	11q del
**CLL 15**	75	M	B/II	No	91	UNMUT	UNMUT	UNMUT	–	11q del
**CLL 16**	71	M	C/IV	Ibrutinib, chlorambucil, bendamustine	96	UNMUT	UNMUT	UNMUT	–	Normal
**CLL 17**	59	M	B/II	No	95	UNMUT	UNMUT	UNMUT	–	Normal
**CLL 18**	71	M	A/0	No	97	UNMUT	UNMUT	UNMUT	–	Normal
**CLL 19**	58	M	B/II	FCM	95	UNMUT	UNMUT	UNMUT	–	13q del
**CLL 20**	55	M	BII	CHOP	86	UNMUT	UNMUT	UNMUT	–	13q del
**CLL 21**	63	F	BII	Fludarabine, FCM, CHOP	90	UNMUT	UNMUT	UNMUT	–	Normal

*M* male, *F* female, *UNMUT* unmutated, *MUT* mutated, *ND* not determined, *del* deletion, *FCM* fludarabine, cyclophosphamide, mitoxantrone, *CHOP* cyclophosphamide, doxorubicin, vincristine, prednisone, *2CdA* cladribine, *R* rituximab

^a^Quantified by flow cytometry

^b^All the mutations have a frequency of >20%

^c^Assessed by FISH

^d^Sanger sequencing data

### Notch ligand stimulation and cell treatment

Recombinant ligands Jagged1, Jagged2, DLL1, and DLL4 (R&D Systems, Minneapolis, MN, USA) were reconstituted in phosphate buffered saline (PBS). Soluble ligands (10 μg/ml for the experiments in Fig. 1 and 4 μg/ml for the other experiments) were let to bind to 12-, 24- or 96-well flat bottom polystyrene culture plates (Corning Inc., Corning, NY, USA) for 4 h at 4 °C after a spin down. Without removing the excess of soluble ligands, CLL cells were seeded in the previous plates and, after a centrifugation step, the cultures were incubated for the indicated times. Two hours before stimulation with ligands, CLL cells were incubated with the anti-Notch1 antibody OMP-52M51 (kindly provided by Oncomed Pharmaceuticals, Redwood City, CA, USA) at 25 μg/ml or with an isotype control. Stromal cell lines OP9-DLL1 and -DLL4 cells were generated and grown as described [42]. For the generation of OP9 stromal cell lines expressing human Jagged 1 (OP9-JAG1), pLZRS-IRES-eGFP retroviral vectors encoding either human Jag1 and GFP as cell tracer or only GFP were kindly provided by Dr. L. Parreira (Instituto de Histologia e Embriologia, Lisboa, Portugal). Jag1 and GFP retroviral constructs were lipofected (Fugene6, Roche, Basel, Switzerland) into 293T Phoenix-Amphotropic cells and transfected cells were selected with 2.5 mg/ml puromycin (Sigma-Aldrich, St. Louis, MO, USA) during two weeks. Mouse OP9 BM stromal cells (American Type Culture Collection; ATCC CRL-2749) were transduced by centrifugation in the presence or 8 mg/ml polybrene. Forty eight hours post transduction, OP9 cells were analyzed for GFP expression by flow cytometry and sorted for homogeneous high expression of GFP. Quantification by flow cytometry of the levels of JAG1, DLL1, and DLL4 confirmed that 100% of OP9-transfected cells expressed the corresponding ligand (Supplemental Fig. 2).

For stroma cocultures, OP9 cells were plated overnight, and then medium was replaced by CLL cells (2 × 10^6^ cells/mL) previously treated with OMP-52M51 for 2 h, or with untreated cells. After 24 h of coculture, CLL cells were collected by carefully rinsing the wells without disturbing the stroma monolayer and processed as required.

### Protein analysis

Protein extracts were obtained and processed by western blot as previously described [43]. For protein immunodetection, the following specific primary antibodies were used: cleaved Notch1 (Cell Signaling Technology, Danvers, MA, USA) and β-actin (Sigma-Aldrich). Anti-rabbit and anti-mouse horseradish peroxidase (HRP)-labeled IgGs (Cell Signaling Technology) were used as secondary antibodies. Chemiluminescence was detected with ECL substrate (Pierce Biotechnology, Rockford, Il., USA) on a mini-LAS4000 Fujifilm device (GE Healthcare). Signal was quantified with Image Gauge densitometric software v4.0 (Fujifilm, Tokyo, Japan) and referred to the respective control.

### Quantitative real-time PCR

Total RNA was isolated from CLL cells as previously [11]. cDNA was obtained from 0.5–1 μg of DNA-free RNA with the M-MLV reverse transcriptase (Thermo Fisher Scientific, Waltham, MA, USA). Samples were processed to Specific Target Amplification using the Fluidigm PreAmpMaster Mix (Fluidigm Corporation, San Francisco, CA, USA) and the following TaqMan Gene Expression Assays (Thermo Fisher Scientific): *HES1, MYC, DTX1, CCND1, NRARP, NPM1, CXCR4*, and *VEGFA*. The relative expression of each gene was quantified by the comparative cycle threshold (Ct) method (ΔΔCt), using *GUSB* as endogenous control. To statistically address the effect of DLL4, no-ligand control condition was used as a reference, and to address the effect of OMP-52M51 on DLL4 stimulation, OMP-52M51 plus DLL4 condition was compared with DLL4 control condition.

### Immunohistochemistry and confocal microscopy

Lymph node biopsies from CLL cases were obtained from the Hematopathology collection. Formalin-fixed paraffin-embedded tissue slides (serial 8 μm sections) were deparaffinised in xylene and etanol-graded series. Tissue antigens were retrieved by boiling during 10–15 min in sodium citrate (10 mM, pH 6.0) and slides were allowed to cool down to room temperature (RT), and then washed in distilled water and PBS. Quenching and permeabilization were carried out using 1% H_2_O_2_ in 100% methanol (40 min, RT) and 0.3% Triton-X-100 (20 min, Sigma-Aldrich) in PBS, respectively. Sections were incubated overnight with the following primary antibodies: anti-human DLL4 (Rabbit, Santa Cruz, Dallas, TX, USA) and anti-human CD68 (Dako, Glostrup, Germany). Background staining was determined by incubating with irrelevant antibodies. Unspecific fluorescence was quenched by incubating with avidin/biotin blocking solutions (Vector Lab, Burlingame, CA, USA). For DLL4 detection, tissue slides were incubated with anti-rabbit IgG-HRP antibody (in 5% BSA in PBS, Dako) and then, the signal was amplified by incubation with the Cyanine-3 Tyramide Signal Amplification Kit (TSA; NEL 744, Perkin Elmer, Waltham, MA, USA). For CD68 immunodetection, a biotinylated anti-mouse IgG (Vector Lab) was added following incubation with avidin/biotin complex (Elite Vectastain ABC Complex kit, Vector Lab). Next, signal was developed by adding Alexa-488-conjugated streptavidin. Nuclei were stained with Topro-3 (Invitrogen, Carlsbad, CA, USA) and mounted with Fluoromount-G (Southern Biotech., Birmingham, AL, USA). Images were acquired using a LSM510 laser scan confocal microscope (Zeiss, Oberkochen, Germany) coupled to an Axiovert 200 (Zeiss) microscope, using ×63 Plan-Neofluar magnification. Images were processed using ImageJ and Adobe Photoshop CS3 software.

### CFSE-based proliferation assay

CLL cells were stained with carboxyfluorescein diacetate succinimidyl ester (CFSE; Thermo Fisher Scientific) as described [16] with some modifications. Briefly, cells at 10 × 10^6^/ml were incubated for 10 min in the dark with 0.5 μM CFSE. Then, an equal volume of FBS was added for another 10 min and the cells were washed twice. CFSE-labeled CLL cells were cultured in an enriched RPMI 1640 medium used for long-term cultures. Specifically, this enriched medium contained 15% heat-inactivated FBS, 1X ITS (insulin, transferrin, selenium cocktail; Biowhittaker, Walkersville, MD, USA), 1 M pH7.3 HEPES, 1X nonessential aminoacid solution (Thermofisher Scientific), 100 mM sodium pyruvate, 1X 2-mercaptoethanol (Thermofisher Scientific) and 50 mg/mL gentamicin sulfate (Biowhittaker). Recombinant human IL-15 (R&D Systems) and the TLR9 ligand CpG oligonucleotide (ODN-2006; Invivogen, San Diego, CA, USA) were added at final concentrations of 15 ng/ml and 0.2 μM, respectively. After 6 days of incubation, cells were collected and stained with Annexin-V-Pacific Blue (PB; Thermo Fisher Scientific) and CD19-PE (Becton Dickinson, Franklin Lakes, NJ, USA) and acquired in an Attune acoustic cytometer (Thermo Fisher Scientific). CLL proliferation was measured as the reduction on CFSE fluorescence based on the median fluorescence intensity (MFI) on the CD19+ and Annexin-V negative cell population. Data analysis was performed using FlowJo v10.0.7 software (FlowJo, Ashland, OR, USA).

### CXCR4 staining

CLL cells were stained with the Live/Dead® Fixable Aqua Dead Cell Stain Kit (Life Technologies) and blocked with 10% mouse serum (Sigma). Then, samples were CXCR4-PE or mouse IgG1 isotype- PE (Becton Dickinson) labeled and acquired in an Attune acoustic cytometer (Thermo Fisher Scientific). CXCR4 expression levels are showed based on the MFI on viable cell population, using untreated cells as a reference.

### Chemotaxis assay

After 48 h of incubation with Notch ligands and OMP-52M51, CLL cells were washed twice and serum starved for 1.5 h in FBS-free RPMI (10^7^ cells/ml). One hundred μl of diluted cells (5 × 10^6^ cells/ml with 0.5% BSA in RPMI) were added to the top chambers of transwell culture polycarbonate inserts with 6.5 mm diameter and 5 μm pore size (Corning Inc.). Inserts had been previously transferred to wells containing 600 μl of 0.5% BSA in RPMI with 200 ng/ml of CXCL12 or 500 ng/ml of CXCL13. Total cell count was obtained from adding 100 μl of cell suspension to wells containing 600 μl of 0.5% BSA in RPMI. After 4 h, 100 μl were collected in triplicate from each lower chamber and total wells, and viable cells were gated and counted on an Attune cytometer (Thermo Fisher Scientific) under constant flow rate. Migration was represented as percentage of migrating cells out of total viable cells added to the transwell.

### HUVEC tube formation assay

HUVEC cells, kindly provided by Dr MC Cid (IDIBAPS), were cultured as previously described [44]. Supernatant from CLL cells (2 × 10^6^ cells/ml) was harvested after 72 h of incubation with OMP-52M51 and ligand stimulation. Twenty-four-well plates were plated with 300 μl of Matrigel (Becton Dickinson). Subsequently, 500 μl of the supernatant of interest and 500 μl of HUVEC cells (0.8 × 10^5^ cells/ml) were added into each culture well. For the control, the supernatant was substituted for complete RPMI medium. After 24 h of incubation, the number of branch points was counted and quantified as the mean of five randomly chosen fields from each well. Pictures were taken at ×40 magnification in a DM IL LED microscope (Leica, Solms, Germany) coupled to a DFC295 camera with Leica Application Suite v3.7 software (Leica).

### Statistical analyses

Data are represented as mean ± SEM of all cases. Statistics were calculated using GraphPadPrism 4.0 software (GraphPad Software, La Jolla, CA, USA). Nonparametric Wilcoxon signed rank test was used to compare the median of a set of samples to a theoretical value. Comparison between means of two sets of samples was evaluated by nonparametric Wilcoxon matched-pairs test or Mann–Whitney test. Results were considered statistically significant when *p*-value < 0.05 (*p < 0.05, **p < 0.01, ***p < 0.001).

## Supplementary information

Supplemental Data
