# Supplementary material for: Specific NOTCH1 antibody targets DLL4-induced proliferation, migration, and angiogenesis in NOTCH1-mutated CLL cells
Source: Oncogene. 2019 Oct 15;39(6):1185–97. doi: 10.1038/s41388-019-1053-6 (PMC7002297; doi:10.1038/s41388-019-1053-6)
Supplement: Supplementary file 1 — Supplemental Data [file 41388_2019_1053_MOESM1_ESM.pdf]

Supplemental Figure 1

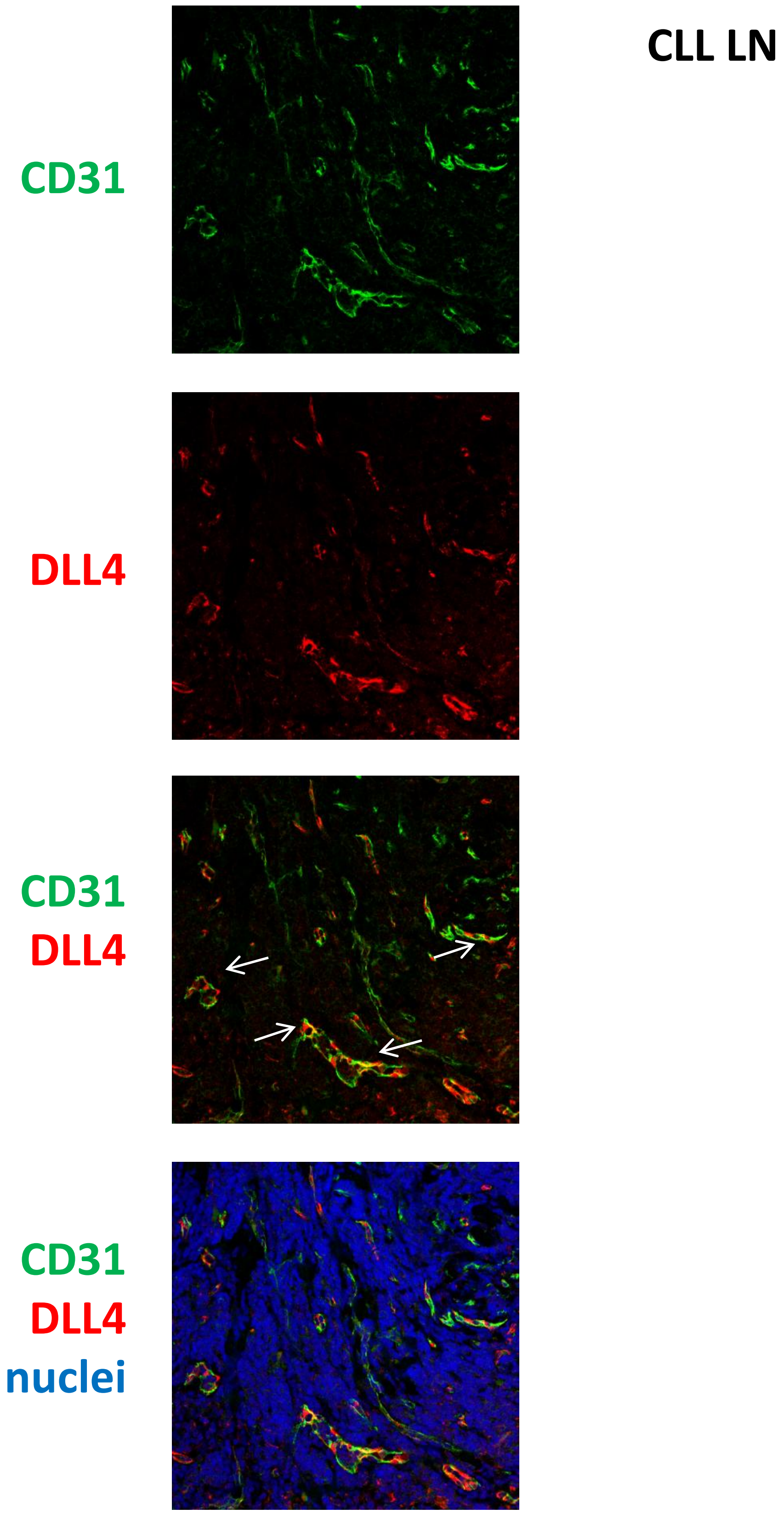

Supplemental Figure 1. Immunofluorescence staining of CLL LN with anti-CD31 (green) and anti-DLL4 (red). Nuclei were stained with Topro-3 (blue). White arrows indicate representative CD31+/DLL4+ cells. Pictures were taken at 25x magnification in a confocal microscope. Images from a representative case are shown.

Supplemental Figure 2

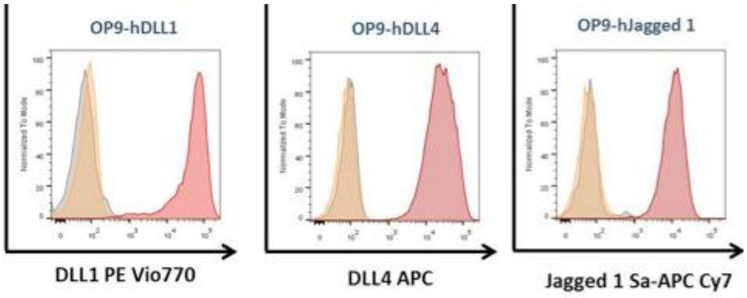

Supplemental Figure 2. Levels of hDLL1, hDLL4 and hJagged 1 in OP9 cells analyzed by flow cytometry.
